# Evaluation of a novel cloud-based software platform for structured experiment design and linked data analytics

**DOI:** 10.1038/sdata.2018.195

**Published:** 2018-10-03

**Authors:** Hannes Juergens, Matthijs Niemeijer, Laura D. Jennings-Antipov, Robert Mans, Jack Morel, Antonius J. A. van Maris, Jack T. Pronk, Timothy S. Gardner

**Affiliations:** 1 Department of Biotechnology, Delft University of Technology, Van der Maasweg 9, 2629 HZ Delft, The Netherlands; 2 Riffyn, Inc., 360 17th Street, Suite 100, Oakland, CA 94612, USA

**Keywords:** Databases, Microbiology techniques, Research management, Research data, Industrial microbiology

## Abstract

Open data in science requires precise definition of experimental procedures used in data generation, but traditional practices for sharing protocols and data cannot provide the required data contextualization. Here, we explore implementation, in an academic research setting, of a novel cloud-based software system designed to address this challenge. The software supports systematic definition of experimental procedures as visual processes, acquisition and analysis of primary data, and linking of data and procedures in machine-computable form. The software was tested on a set of quantitative microbial-physiology experiments. Though time-intensive, definition of experimental procedures in the software enabled much more precise, unambiguous definitions of experiments than conventional protocols. Once defined, processes were easily reusable and composable into more complex experimental flows. Automatic coupling of process definitions to experimental data enables immediate identification of correlations between procedural details, intended and unintended experimental perturbations, and experimental outcomes. Software-based experiment descriptions could ultimately replace terse and ambiguous ‘Materials and Methods’ sections in scientific journals, thus promoting reproducibility and reusability of published studies.

## Introduction

Iterative progress in science and technology rests on the fundamental assumption that researchers can build off each other’s published findings. Turning this assumption into reality can, however, be far from trivial for any field of science^1^. Accurate descriptions of experimental procedures are essential for meaningful comparison, interpretation and re-use of data acquired in different laboratories, at different times, and/or by different researchers. However, Materials and Methods sections in scientific journals are extremely terse, leaving room for subjective interpretation, and are often insufficient to accurately reproduce the stated findings. The magnitude of this challenge is illustrated by the frequent occurrence and estimated costs of irreproducibility in multi-laboratory studies in the life sciences^2–4^, and the emergence of programmes that fund replication of impactful research, such as the Dutch Replication Studies pilot^5^.

Problems related to reproducibility and reusability of experimental data are further compounded by incomplete sharing of primary data and/or disconnection of data from the exact experimental context in which they were generated. For example, microbial biotechnology publications rarely include complete sets of primary data obtained in controlled-cultivation experiments (e.g., actual concentrations of metabolites in culture samples). Instead, publications generally only report derived parameters (e.g., biomass-specific conversion rates and yields) which might even be calculated differently by different researchers. Conversely, genome-wide ‘omics’ data derived from cultivation experiments are routinely deposited in publicly accessible repositories, yet the description of the exact experimental context in which data were generated is often limited and disconnected from the data. For example, transcriptome datasets generally only contain incomplete descriptions of experimental procedures, even though transcriptome analyses are notoriously sensitive to minor differences in experimental conditions, such as different sources of chemicals, cultivation procedures, and/or equipment settings^6,7^.

For a dataset to be truly reusable, all experimental data must be directly linked, in computable form, to the methodological information used in data generation. Data is considered to be in a “computable form” when each data point is annotated or linked to standardized data describing its experimental context and structured into standard formats suitable for data processing. Such data displays clear relationships between the variables and the observations (typically as rows and columns in a standard statistical data table) that allows search, quantitative filtering, categorization, summarization and correlation of data by visualization software (e.g., Spotfire, Tableau), statistical software (e.g., R, SAS, JMP) or automated data pipelines (built, for example, on Python or Apache Spark). This format allows for meaningful re-interpretation and re-use of data through human or algorithmic analysis of data integrity and scientific validity. Computable, contextualized data sets also permit large-scale search and aggregation of data for use in increasingly powerful machine learning technologies.

Several scientific journals, including Scientific Data, are at the forefront of the drive towards open data publishing and data reusability^8^. Yet, these much-needed efforts are currently limited in their impact because tools that automatically link procedural information to measurement data in computable form are practically non-existent. General-purpose data repositories, e.g. FigShare (figshare.com), Dryad Digital Repository (datadryad.org), Mendeley Data (data.mendeley.com) and Zenodo (zenodo.org), facilitate access to data files via the cloud, but do not require computable methodological information to be published alongside datasets. Software like ISA Tools^9^ allow researchers to annotate datasets with metadata, thus increasing data discoverability. However, such procedures require extensive human annotation of data sets *after* data collection.

In 2016, Riffyn launched a new cloud-based software, the Riffyn Scientific Development Environment (SDE), for computer-aided design and analysis of experiments, with the goal of addressing challenges in data contextualization and reusability. Here, we evaluate this software in the practical setting of academic microbial biotechnology research, and discuss both its advantages and challenges for experiment design, data contextualization, and reuse of results in academic research. The evaluation was undertaken via a collaboration between scientists in the Industrial Microbiology section of the Delft University of Technology (TU Delft, the “evaluation team”), and scientists at Riffyn who support the Riffyn SDE application (the “support team”). The Riffyn SDE was used by the evaluation team, in their laboratory at TU Delft, to document, execute and analyze microbial cultivation in shake flasks, batch bioreactors and chemostat bioreactors.

**Figure 1:**
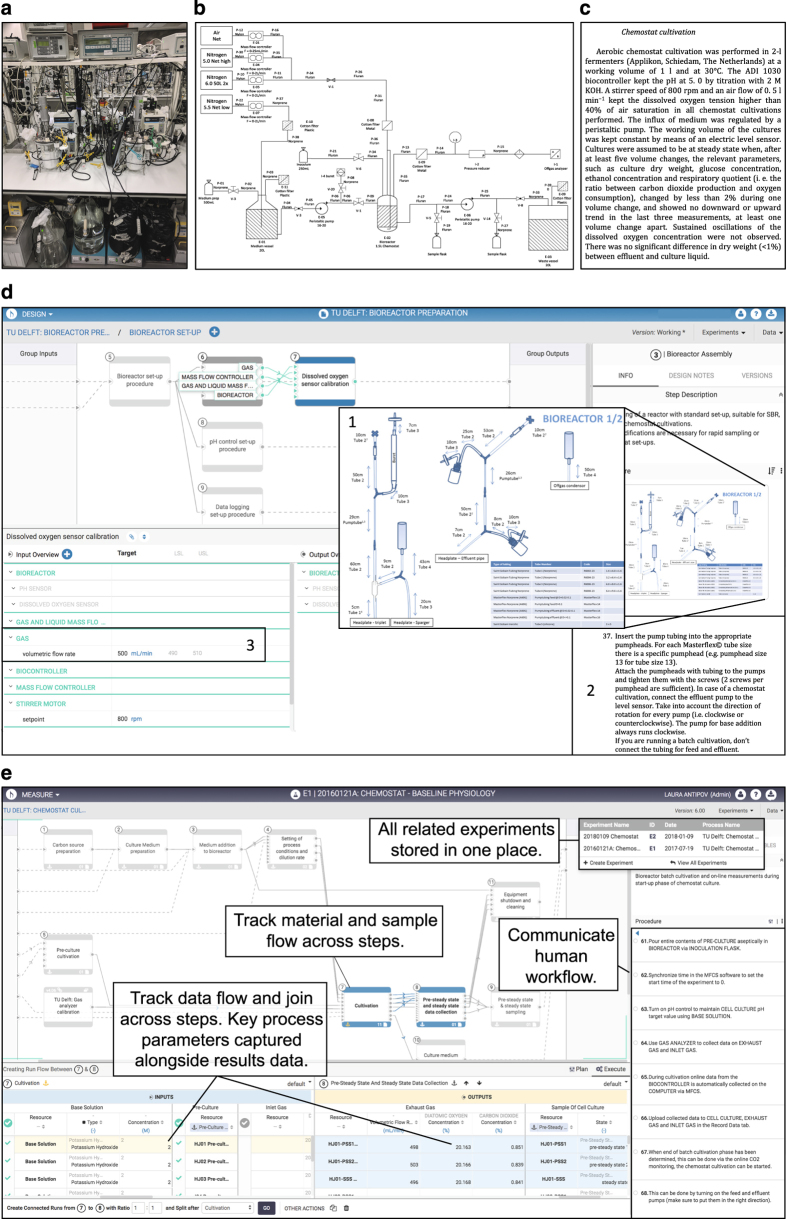
Use of the Riffyn SDE to transform traditional documentation of an experimental procedure into visual process flows and annotated, computable experimental datasets. (**a**) Photograph of a bioreactor setup for chemostat cultivation, which combines equipment for aseptic liquid/gas handling and maintenance of a stable environment for microbial cultures, as well as measurement and data logging devices. (**b**) Flowsheet that might be used to communicate a bioreactor setup to other researchers, containing information about the type of mechanical bioreactor components used. (**c**) A typical text describing chemostat cultivation in a Materials and Methods section of a scientific publication, outlining the conditions and settings most important to the experiment. (**d**) The Riffyn SDE’s Design Mode allows the capture of illustrations to demonstrate complex bioreactor set-ups (d1), as well as detailed step-by-step instructions for such setups (d2), and target parameter values and their upper and lower spec limits for specific bioreactor settings (d3). (**e**) The Riffyn SDE’s Measure Mode allows the association of data with process design backbones. Material, equipment and sample flow across steps are tracked; data associated with these entities are also tracked and joined across steps. Key process parameters, traditionally captured in lab notebooks, are captured electronically alongside results data otherwise captured in instrument files or spreadsheets. The human workflow needed to execute the experiment, traditionally recorded in lab notebooks or by word of mouth is recorded in context on each step. Also illustrated in this panel (upper right) are multiple experiments captured on this same process which will be collated together automatically during data analysis.

Bioreactor cultivation offered a suitable use-case for the evaluation because it is a complicated, technically involved process (Fig. 1a-c) that is presently documented with lengthy prose descriptions of the procedural steps and parameter settings involved. These documents are difficult to maintain and therefore frequently become out-of-date due to continuous evolution and improvement in cultivation methodologies. Moreover, even detailed protocols used within research groups can fail to fully capture experimental procedures, leading to confusion about how data were collected, and thus impairing future data analysis or quality assessment. The evaluation team, therefore, sought to assess if the Riffyn SDE would offer a meaningful improvement in the design, operation and analysis of such microbial biotechnology experiments.

### Riffyn Scientific Development Environment (SDE)

The Riffyn SDE is a cloud-based software system for computer-aided design of experiments and data analysis. It is designed to enhance experimental data reproducibility and reusability by providing automatic data contextualization, and structured, computationally mineable, reusable datasets.

Data contextualization occurs in the Riffyn SDE by capturing data in three different flows: (1) material and equipment flow, (2) human workflow, and (3) data flow (Fig. 1d-e). This data capture is achieved by transforming experimental protocols into reusable templates called “processes”. Processes are created in the Riffyn SDE’s “Design Mode”, wherein experimental procedures are recorded as stepwise process flow diagrams, and the flow of materials and equipment across steps is systematically tracked. All relevant settings and key defining characteristics of these materials and equipment are specified directly in the process design. To communicate the human workflow needed to complete an experiment, detailed procedural instructions are also associated with each step of the process.

Each process can then be copied as one or more experiments, which are executed in the Riffyn SDE’s “Measure Mode”. Each of these copies reflects and preserves the version of the process at the time it was created, and becomes the computable backbone to which the experimental parameters, sample identifiers, and data are attached. Because experimental data and procedural parameters are recorded on the same backbone, measurement data (such as metabolite concentrations) get automatically and permanently joined to the protocol variables (such as HPLC mobile phase) used to obtain those data. Thus, experimental data are automatically contextualized, eliminating the need to annotate datasets with metadata after the experiment is complete.

**Figure 2:**
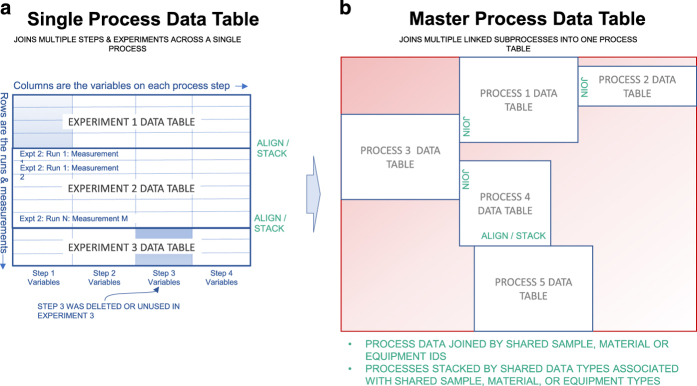
Structure of an experimental data table produced by the Riffyn SDE, and its assembly into larger aggregate data tables for data analysis and machine learning. (**a**) All data on each experiment in the Riffyn SDE is extracted and flattened into a statistical data-frame compatible with nearly any modern analysis software (including R, JMP, SAS, Tableau, Minitab, etc.). This data-frame is composed of all variables defined on a Riffyn SDE process or experiment, including sample identifiers, parameter settings, start/stop times, dates, measurement data and units. The variables are presented as columns in the data-frame and grouped by step in the process. Multiple experiments executed on that same process (regardless of version) are stacked together such that data are aligned into common columns. If versions of a process diverge, gaps are left in the table where such variables are missing in the process. (**b**) Individual process data tables can be further combined into a “Master Process” data table composed of all data from all linked processes used in performing an experiment, including material preparation, equipment set-up, fermentation, and analytical chemistry processes. Data from these processes are joined together using the material flow graph defined by the Master Process in the Riffyn SDE, and the material identifiers automatically tracked by the Riffyn SDE.

After annotation with procedural information, the Riffyn SDE automatically joins the data across processing steps to provide the full provenance and traceable genealogy of materials, samples and reagents used as inputs to the experiments (Fig. 2). The data joining performed by the Riffyn SDE is distinct from traditional data linking, which is often in the form of hyperlinks. In the Riffyn SDE, a join produces a standard statistical data frame ready for immediate visualization, statistical analysis and machine learning in common software such as Python, R and JMP. The Riffyn SDE joins data in three ways: (1) “horizontally” across all steps in a process flow diagram, (2) “vertically” across multiple experiments executed on a process (data concatenation), and (3) across multiple processes connected by shared sample outputs and inputs. The result is a comprehensive statistical data table containing all methodological and experimental data collected on a process or group of processes. This data table is searchable and computationally mineable, enabling immediate analyses of data quality, correlations, time-series analysis, causal relationships, and root causes of error.

Although bioreactor experiments were used as a test case in this study, the Riffyn SDE is not restricted to this domain of science. The Riffyn SDE has to-date been used for a variety of experiments including enzyme assays, HPLC assays, genetic engineering protocols, compound and material formulations studies, material preparation processes, cell line screening, animal pharmacokinetics, drug tablet manufacturing process development, food processing, and chemical or protein recovery and purification processes. Examples of some of these processes are provided as public processes on the Riffyn cloud at http://app.riffyn.com.

### Riffyn SDE System architecture

The Riffyn SDE is a cloud-based software-as-a-service architecture hosted and operated by Riffyn in both multi-tenant and single-organization configurations on Amazon Web Services. Its graphical user interface is accessed manually via a web browser, or programmatically via a web API. The system is built on a foundation of NodeJS and Python language with the MeteorJS framework, SVG, D3 and WebGL graphics, OpenSSL encryption, and NginX load balancing. The Riffyn SDE software automatically saves all edits and data uploads, and concurrently updates all active web browsers such that users can see each other’s changes in real-time. All data remains encrypted throughout the system, both in transit and at rest using TLS 1.1 and 1.2 protocols over https and websockets with a variety of cipher keys. Data is stored in a MongoDB with three-fold real-time failover protection, a fourth real-time replica database, and static full-database backups every 6 hours, which are saved for 6 months to an offsite location for disaster protection. Data storage provides assured consistency on the primary member of the replica set, with eventual consistency to the secondary members of the replica set. The system automatically distributes load over a minimum of 18 servers with three-fold redundancy, and is horizontally scalable as load increases over time. Multimedia is stored in Riffyn as raw (original) files on AWS S3 buckets, ensuring scalability.

Data captured in the Riffyn SDE is automatically parsed, atomized and stored in a flexible No-SQL database as JSON document objects, using a hypergraph data model that links all data to the material and equipment sources that generated it^10^. The Riffyn SDE integrates standard scientific ontologies from public sources such as BioPortal^11^, ensuring standardization of terminology used to annotate experiments. The data model also allows for the capture of single-point data (e.g. microtiter per-well measurements), time series data (e.g. batch bioreactor data) and event series data (e.g. chromatography spectra and flow cytometer data) in the same underlying data structures. When data is requested by the user, the Riffyn SDE analytics engine walks the underlying material hypergraph, automatically annotates measurements with experimental metadata, links them based on source/child material genealogy, and reshapes them into the flat datatables suited for immediate visualization and statistical analysis.

**Figure 3:**
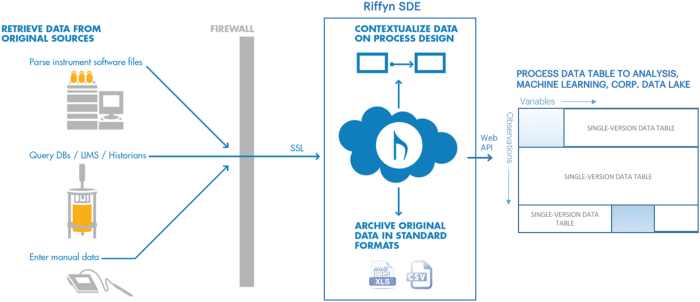
Schematic representation of data capture, data archival, and data joining performed by the Riffyn SDE. Data is entered into Riffyn via parsing of instrument files, querying of a database, or via manual data entry. All data is passed through a firewall and contextualized based on the Riffyn SDE process design. Any files parsed during the data upload are additionally stored in their native format. All contextualized data is exported as a flat csv file with all data, material information, and setpoints captured. This flat file is built with each variable as a column, and each row as an observation. Multiple tables are concatenated together across each version of the process to provide a comprehensive, cross-version table that captures all data across the development of the process.

Data exported from the Riffyn SDE is provided in vendor-neutral CSV, JSON or XML format and provides a complete description of both experimental method and measurement data, such that users are not bound by proprietary data formats or storage. All data can be streamed in real-time to external data stores, maintained independently of the Riffyn hosting service. A schematic representation of this data capture, archival and joining is show in Fig. 3.

Riffyn is capable of handling large amounts of data on each experiment and each sample collected. Prior examples include writing 10,000 measurements to a single sample in some long-running temporal experiments, and collecting and tracking 10,000 samples in a screening experiment. Riffyn is currently extending its capabilities to permit tracking of 100,000 samples per experiment (by autumn 2018), but high-throughput screening experiments of a larger scale (millions of samples per experiment) require more specialized automation software.

### Experiments performed in this study

To evaluate the ability of the Riffyn SDE software to capture and communicate complex protocols, record diverse data types, and enable data analysis, sharing, and reusability, we applied it to a study to quantify the aerobic growth characteristics of the industrially relevant yeast *Ogataea polymorpha* (*syn. Hansenula polymorpha*). This methylotrophic and thermotolerant yeast is used at an industrial scale for biotechnological production of recombinant proteins^12^, and has several interesting properties for application in other processes. These properties include an ability to grow at temperatures up to 50°C, consumption of a wide range of substrates, low byproduct formation in aerobic cultures and a generally recognized as safe (GRAS) status^13,14^. Despite its industrial applications, and many academic studies of its growth on methanol^15,16^ and nitrate^17,18^, aerobic growth characteristics of *O. polymorpha* on glucose have not been described comprehensively. Here, the aerobic physiology of two popular *O. (para)polymorpha* strains (CBS4732 and CBS11895/DL-1) was investigated in a glucose-containing synthetic medium^19^. In a first set of experiments, shake flask cultures were used to determine specific growth rates over a range of temperatures. Subsequently, a precise quantitative analysis of growth kinetics was performed in both bioreactor batch cultures and chemostat cultures.

In shake flask cultures, cells were added to sterile synthetic growth medium and incubated aerobically at temperatures ranging from 30 to 49°C. Optical density measurements were performed at regular intervals to enable calculation of the maximum specific growth rate at each temperature. Subsequently, both strains were characterized in bioreactor batch cultures at 30°C (often used for characterization of yeasts) and 40°C (optimum growth temperature determined in shake flasks). Bioreactor batch cultivation allowed for control of medium pH, continuous off-gas measurement, as well as intensive mixing and gas transfer to prevent oxygen limitation of fast-growing microbial cultures. Samples from the bioreactor cultures were used to determine biomass concentration and analyzed by HPLC to quantify the extracellular metabolite concentration. Finally, both strains were grown in chemostat cultures. In a chemostat, simultaneous addition of fresh medium to the bioreactor and removal of spent culture broth occurs at a fixed dilution rate D [h^-1^], defined as the outgoing liquid flow F_out_ [L h^-1^] divided by the liquid culture volume V_L_ [L] in the reactor (D = F_out_/V_L_ [h^-1^]). When D does not exceed the highest specific growth rate μ [h^-1^] that the organism can reach under the experimental conditions, a nutrient-limited steady state will ensue in which μ equals the dilution rate set by the experimenter^20^. In this study, the physiology of both *O. (para)polymorpha* strains was compared at 30 and 40°C in steady-state chemostat cultures operated at D = 0.10 h^-1^.

## Results

### Experiment design in Riffyn SDE Design Mode

The Riffyn SDE’s Design Mode was evaluated by applying it to microbial cultivation in shake flasks and bioreactors, the latter in batch as well as chemostat mode.

Design Mode was used to define a process comprising detailed, step-by-step procedural instructions, visualized by process flow diagrams in which all required materials and equipment are specified along with relevant settings and other process parameters. For example, the process for bioreactor cultivation includes detailed descriptions of the composition and preparation of synthetic growth media and of pH setpoints and control during cultivation. Moreover, steps that are difficult to explain in written protocols, can be clearly defined by embedding videos or photographs into the Riffyn SDE process diagram. For instance, the procedure for linking two stainless-steel connectors during coupling of a new medium reservoir to a bioreactor, which is difficult to describe in words but essential for aseptic operation of chemostat cultures, was adequately described by a short video embedded on the corresponding step 3 of the Process (https://app.riffyn.com/processes/24K9JeAQg6vJHPrg5, see Methods section for access to Riffyn SDE).

Although definition of a process involved significant time investments, this activity encouraged the evaluation team to critically assess and discuss all individual steps in an experimental procedure and describe them unambiguously. For example, definition of a process for chemostat cultivation inspired more rigorous definition and/or standardization of the length and diameter/type of tubing used on bioreactors, the procedure for setting pump-speeds for chemostat setups, the dead volume of sampling ports, and the treatment of off-gas before analysis. These insights were put to good use when, in the midst of the software evaluation, a physical move of the laboratory to a new building necessitated the complete disassembly and reassembly of all its 40 bioreactor set-ups. At a deeper level, the design phase, which precedes and defines the actual execution of the experiments, compelled researchers to think critically about which experimental parameters to collect and record. This intrinsic link between experimental design and execution allows users to improve interpretability of experiments. For example, the procedures in the inoculation phase, an essential step in microbial cultivation that involves experimental parameters such as volume, biomass concentration, physiological status and means of addition of a preculture, can drastically affect experimental outcome but are rarely reported in literature in a completely unambiguous manner.

The Riffyn SDE software automatically tracks changes to protocols and allows generation of a new version at any time. This ensures that the most up-to-date and/or most suitable variant of a process is available to experimenters, thereby reducing the amount of potential errors and mistakes. Change tracking also ensures that (subtle) changes in an experimental process can be correlated retrospectively to changes in experimental outcome.

### Data collection in Riffyn SDE Measure Mode

The processes defined in Design Mode were used as templates for data collection in Measure Mode to characterize aerobic growth of *O. polymorpha* on glucose in shake flasks and in bioreactor batch and chemostat cultures. In the evaluation team’s laboratory, data are usually recorded in laboratory notebooks and later combined in Excel for calculation of key experimental outcomes. By contrast, in Measure Mode, the experimenter is systematically guided through each step of the experiment and prompted to collect parameter values (defined in Design Mode), such as equipment settings, amounts of reagents used and associated experimental measurement data (e.g. on-line off-gas measurements and metabolite/biomass concentrations) in real time.

In contrast to the classical use of lab journals, where data are usually recorded on a ‘by date/time’ basis, the Riffyn SDE’s Measure Mode stores all measured data for a particular experiment together. This is especially relevant and helpful when cultures are tracked over time (e.g. batch cultivation experiments that cover multiple days), where the primary recorded data often become fragmented when multiple experiments are performed in parallel. Via a built-in plotting tool, newly obtained measurements could be directly integrated with data obtained at earlier time-points. This feature could be very useful for identifying sampling or analysis errors on the spot, which could prompt the experimenter to take another sample.

A useful feature of Measure Mode was the option to make changes to process designs while performing an experiment. This possibility is especially valuable when an experimenter is confronted with an unexpected development, such as a sudden change of supplier of a chemical or the need to freeze and store samples because analytical equipment is malfunctioning. Since the Riffyn SDE software unambiguously links any such modifications in the process design to the relevant data, their potential impacts on experimental outcomes remain unambiguously documented and can be retrospectively identified.

### Data analysis

In the evaluation team’s laboratory, students and employees typically manually extract and combine relevant data generated in their experiments, often into individually prepared spreadsheets, to perform calculations and compare experimental outcomes. This approach requires constant checks for consistency and accuracy, which can be inconvenient and time-consuming when applied to large experiments and datasets. The limitations of this approach become especially evident when checking and/or integrating data obtained at different times, by different researchers, and/or in different laboratories. Recording process parameters and experimental data with the Riffyn SDE Design and Measure Modes streamlined and standardized data capture and analysis. The software automatically generated CSV-formatted data tables for each process, combining not only the process parameters and experimental data for each step, but also from other experiments that were conducted using a specific process (Fig. 2).

**Figure 4:**
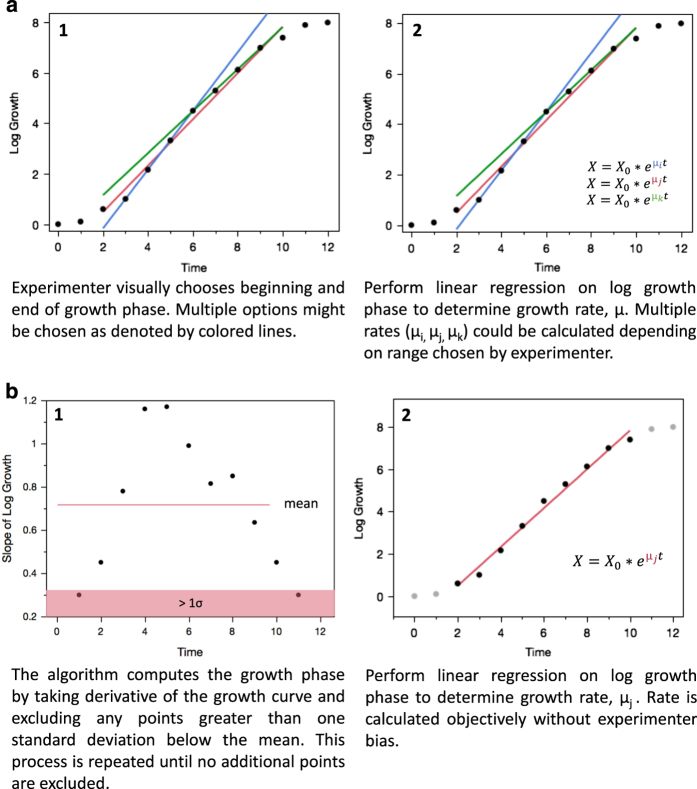
Comparison of analysis methods used for calculations of growth rates and other physiological parameters. (**a**) The ad hoc manual method typically used by experimenters requires visual inspection of the growth curves to exclude data points believed to be outside of exponential phase. After exclusion of such points, growth rates are calculated by regression of the remaining data points. This approach is subject to subjective interpretation and human bias. (**b**) The automatic growth rate calculation method implemented in scripts associated with the cultivation processes in the Riffyn SDE. An algorithm, rather than visual inspection, is used to identify values outside the exponential growth phase. Growth rates are then calculated using the remaining data points. Physiological parameters derived from such growth rates are objectively calculated, without human bias.

Even when starting from the same dataset, different researchers may adopt different ways to calculate derived parameters and perform statistical analyses. This problem especially occurs when calculations are based on a manually selected subset of the experimental data. For example, calculations of specific growth rates in microbial batch cultures are based on biomass and/or optical density measurements taken during the “exponential growth phase”, with the exponential growth phase being defined in an ad hoc way by visual inspection of data by each researcher. The Riffyn SDE software resolved this issue of human bias by enabling an objective, automated data calculation pipeline for all physiological parameters (Fig. 4). This pipeline utilized the automatically-generated Riffyn data tables in combination with a JMP software script, both of which are archived together with the experiment in Measure Mode. For example, the specific growth rate calculation script automatically defined the exponential phase of growth curves based on objective criteria, and then computed specific growth rates from those phases. This automated analysis was used to calculate specific growth rates, biomass yields and biomass-specific glucose uptake rates of two *O. (para)polymorpha* strains (CBS4732 and CBS11895) in aerobic bioreactor batch cultures at 30 and 40°C (Table 1) as well as for their quantitative physiological analysis in shake flask and chemostat cultures (Supplementary Tables S1). A summary of the design of the analysis scripts, and links to the full scripts within the Riffyn SDE, are provided in the Methods section.

## Discussion

The Riffyn SDE offers a novel process-based approach to scientific experimentation, with the ability to integrate method definitions, primary research data, and data analysis scripts into a shareable representation of experiments. These visually expressive process descriptions increase the re-workability of complex procedures and the reusability of the data they generate. They are also computable which facilitates their use by search, data mining and data analysis programs. This approach could potentially address an Achilles heel of open data policies in academic research and publishing: the exchange of poorly contextualized, subjectively analyzed, and therefore intrinsically non-reusable data.

This evaluation illuminated some challenges in the initial implementation of the Riffyn SDE into a real-life academic research setting, mostly during first definition of experimental procedures. Firstly, getting acquainted with the software is a substantial up-front time investment for new users. Secondly, design of experiments in Riffyn is more time intensive than writing conventional protocols in prose format, as the designs are more complex and provide the framework for subsequent experimental execution and data analysis within the software. Furthermore, this connection between design, data collection and analysis can lead to the need for multiple design cycles for design optimization, unless the user is very experienced. However, once finalized, the procedures defined in Riffyn provide a definition of the experimental procedures that is more complete and unambiguous than traditional protocols. Therefore, given our experience during the execution and communication of this trial experiment, we speculate that software like the Riffyn SDE could helpfully augment or replace descriptions in methods sections of scientific publications.

One particular unanticipated benefit experienced by the evaluation team related to experimenter awareness. Implementation of intensively-used procedures in the systematic process format led to identification of missing information in established protocols, which invited the experimenters to rethink and discuss experimental design, process descriptions and standardization. Outcomes of these discussions were then memorialized into a shared representation of the process.

The Riffyn SDE also helped to address the challenges of tracking process changes via its version control system. This provides assurance of stable representation of past experimental methods and their resulting data for future analysis. It prevents undesirable situations where the exact experimental procedures have to be reconstructed retrospectively from the notebooks and memories of different researchers. For example, its potential relevance is illustrated by the severe loss of time that the evaluation team incurred several years ago when their regular supplier of antifoaming agent ceased production. Subsequently, over a substantial period of time, replacement antifoaming compounds were found to affect yeast physiology in a context-dependent manner. Had all these experiments been recorded with the Riffyn SDE software, it would have been simple to correlate, for example, biomass yields or growth rates to the type of antifoam used across a large number of experiments.

We also identified a clear trade-off between the level of process detail implemented and the required investments in scientist time. While every aspect of a laboratory procedure that can influence experimental outcome could theoretically be defined in a Riffyn SDE process, it may not be practical from a time and effort perspective to do so. Especially in sparsely staffed academic research settings, we foresee the largest return on investment of effort when applying the Riffyn SDE to processes that are technically involved, generate quantitative results, are intensively used within a group, and whose basic technical methodologies are not subject to frequent change. In such situations, the processes can be reused and modified for new experiments, resulting in time savings and higher-quality data. Bioreactor cultivation of microorganisms and its associated analyses, which were used as a model for this study, provided an excellent fit with this description. On the other hand, the volatile world of genetic engineering may well represent the opposite end of a scale. When new methods and protocols appear on an almost weekly basis, and the quality of outputs can be verified by genome sequencing or other diagnostic tools, rigorous implementation of protocols as Riffyn SDE processes may require too large an investment in time.

In the evaluation team’s laboratory, written protocols for key methods are currently maintained, updated and approved by academic and technical staff members. Keeping these protocols up-to-date, as well as ensuring that the right versions are used by a continually changing population of PhD, MSc and BSc students requires intensive communication and traffic of document versions. Since the Riffyn SDE is cloud-based, updates instantly, and is not limited to a single user at a time, multiple researchers were able to simultaneously and interactively work on the same process or experiment. These real-time sharing capabilities of the Riffyn SDE provide mechanisms to streamline communication, revision and approval of experimental process definitions. Additionally, the Riffyn SDE’s ability to integrate multimedia approaches in process descriptions could provide a valuable tool for training of new students and employees, thereby minimizing loss of experiments and improving laboratory safety.

Finally, we note that the Riffyn SDE encouraged more automated, reproducible, and objective analysis of experimental data produced in the study. By automatically extracting, combining and reshaping both methodological and measurement data for computational analysis, we were able to develop and apply computational scripts directly to the processes and experiments in the Riffyn SDE. Moreover, by compiling data across multiple experiments, the Riffyn SDE facilitates learning from historical data records. We feel that this approach could enable more rigorous outcomes for the evaluation team’s laboratory and ensure more consistent, traceable, and defensible outcomes when shared with the broader research community.

## Methods

Riffyn SDE experiments serve as the Methods section of this article. Riffyn supports open science and has made all data, methods, code and supporting information available as files in the Supplementary Material (Data citation 1) in both human readable and computable form. In addition, all data are freely available for viewing or downloading via the Riffyn SDE user interface (http://app.riffyn.com) using a web browser, and programmatically accessible via an open application program interface (API) using any software supporting secure http requests (api.riffyn.com). Readers may request (via https://riffyn.com) an open access online account in the Riffyn SDE to view these experiments and create their own processes and experiments. Riffyn provides a copy of its open access policy at https://riffyn.com/riffyn-open-access-policy.

### Riffyn SDE processes

Processes used in this study, including material preparation, cultivation and analytical methods, were composed into an aggregate set of linked processes supporting each type of cell culture modality in this study (shake flask, batch bioreactor and chemostat cultivation). Each set of linked processes is called a “Master Process” (see Fig. 2). Links to each of these Master Processes, and the experiments performed on the sub-processes within them, are provided below for reference. These experiments can also be found by browsing the Master Process structure itself via the Riffyn SDE.

- Shake flask batch cultivation Master Process: https://app.riffyn.com/processes/FonopPptQjMqQMBWm
- Shake flask batch cultivation experiment: https://app.riffyn.com/experiments/ABpJX7NjeQJy9xBTK
- Bioreactor batch cultivation Master Process: https://app.riffyn.com/processes/czj5FuAfazh6if8j2
- Medium preparation experiment: https://app.riffyn.com/experiments/EDuGj2GkQMke6vuEw
- Bioreactor preparation experiment: https://app.riffyn.com/experiments/aqHPvgSFm2apMtGMW
- Bioreactor batch cultivation experiment: https://app.riffyn.com/experiments/BTpb9qytcd5qwPTau
- Biomass dryweight analysis experiment: https://app.riffyn.com/experiments/AnxhXchsrjwpB2zWs
- Metabolite analysis (HPLC) experiment: https://app.riffyn.com/experiments/irhLrZ3s4oCZT7NyC
- Optical density measurement experiment: https://app.riffyn.com/experiments/h6XWEFyNSBmJXrrfS
- Chemostat cultivation Master Process: https://app.riffyn.com/processes/3YstPHztm3kH9nuwX
- Medium preparation experiment: https://app.riffyn.com/experiments/xNojK3JS8LaEdp7aZ
- Bioreactor preparation experiment: https://app.riffyn.com/experiments/ZGXT3PhyvXqFW8bg3
- Chemostat cultivation experiment: https://app.riffyn.com/experiments/GHNJvwkrzeiSdbqme
- Rapid sampling for extracellular metabolites experiment: https://app.riffyn.com/experiments/FntsvnGc838TTepJ7
- Biomass dryweight analysis experiment: https://app.riffyn.com/experiments/qLqiBdFddeZqSE5aC

### Analysis Scripts

Three data analysis scripts used in this study are summarized here. They were each designed to analyze a “Master Process” data table which is a combined data table built by joining all data on a Master Process (see Fig. 2). The Master Process data tables were created using the ‘Get and join experiment data’ function in a JMP Add-In for the Riffyn SDE (JMP Add-In available from https://help.riffyn.com). Each analysis script is stored on the Master Process on the “analysis” step.

**Script 1**: Calculation of results from shake flask batch cultivation data. This script performs a set of successive actions to clean and organize the data before calculating the growth rate of yeast cells based on optical density measurements. The sections of the script, denoted by the line number, are as follows: (1-67) loading, sorting, and cleanup of data; (68-173) calculation of growth parameters and rates; (174-403) summarization and tabulation of results.

**Script 2**: Calculation of results from batch bioreactor cultivation data. This script performs a set of successive actions to clean and organize the data before calculating basic physiological parameters. The sections of the script, denoted by the line number, are as follows: (1-75) loading, sorting, and cleanup of data; (76-328) calculation of growth parameters and rates; (329-681) summarization and tabulation of results.

**Script 3**: Calculation of results from chemostat cultivation data. This script performs a set of successive actions to clean and organize the data before calculating basic physiological parameters. The sections of the script, denoted by the line number, are as follows: (1-97) loading, sorting, and cleanup of data; (98-462) calculation of growth parameters and rates; (463-711) summarization and tabulation of results.

### Code availability

The analysis scripts described above are available as .jsl files (‘JMP scripts used for automatic analysis of Riffyn-generated data files’, Data citation 1). The scripts were written for JMP version 13.1 and can also be opened by any plain text editor.

## Additional information

**How to cite this article**: Juergens, H. *et al*. Evaluation of a novel cloud-based software platform for structured experiment design and linked data analytics. *Sci. Data*. 5:180195 doi: 10.1038/sdata.2018.195 (2018).

**Publisher’s note**: Springer Nature remains neutral with regard to jurisdictional claims in published maps and institutional affiliations.

## Supplementary Material

Supplementary Information

## Figures and Tables

**Table 1:** Temperature dependent physiology of two wild type *Ogataea (para)polymorpha* strains in aerobic bioreactor batch cultures.

	**30 °C**		**40 °C**	
	**CBS4732**	**CBS11895**	**CBS4732**	**CBS11895**
μ_max, dryweight_ [h^-1^]	0.45±0.01	0.36±0.01	0.68±0.01	0.63±0.01
	*±0.008*	*±0.006*	*±0.012*	*±0.010*
μ_max, OD660_ [h^-1^]	0.44±0.01	0.36±0.01	0.69±0.01	0.63±0.01
	*±0.001*	*±0.001*	*±0.001*	*±0.001*
Y_X/S_ [g biomass (g glucose)^-1^]	0.51±0.00	0.52±0.00	0.49±0.01	0.49±0.01
	*±0.01*	*±0.01*	*±0.01*	*±0.01*
q_Glucose_ [mmol (g biomass)^-1^ h^-1^]	-4.90±0.02	-3.90±0.09	-7.77±0.30	-7.15±0.06
	*±0.15*	*±0.15*	*±0.15*	*±0.15*
Reported values are Mean±MAD. Standard errors are reported in italics on the second line.				
Cultures were grown at pH 5 in synthetic medium with an initial glucose concentration of 7.5 g L^-1^. Means and mean absolute deviations (MAD) were calculated from two individual cultures and the standard error was calculated from the pooled estimate of variance across all culture samples from each strain. For some properties subject to multiplicative error (e.g., growth rate), logarithmic transformations of the data were applied to regularize the variance prior to pooling. Data was transformed back to standard linear scale prior for reporting the errors. Symbols: μ_max, dryweight_ = maximum specific cell growth rate based on measurement of biomass dryweight; μ_max, OD660_ = maximum specific cell growth rate based on measurement of culture optical density at 660 nm, Y_X/S_ = yield of cell biomass dryweight on sugar carbon source in the exponential phase, q_Glucose_ = biomass-specific glucose uptake rate in the exponential growth phase.				
