# Supplementary material for: Evaluation of a novel cloud-based software platform for structured experiment design and linked data analytics
Source: Sci Data. 2018 Oct 3;5:180195. doi: 10.1038/sdata.2018.195 (PMC6169258; doi:10.1038/sdata.2018.195)
Supplement: Supplementary Information [file sdata2018195-s1.docx]

# Supplementary information

## Table S1: Temperature dependent physiology of two wildtype *Ogataea (para)polymorpha* strains grown in synthetic medium at pH 5 in aerobic glucose-limited chemostat cultures. Means and mean absolute deviations (MAD) were calculated from two individual cultures and the standard error was calculated from the pooled estimate of variance across all culture samples from each strain. Carbon recovery calculations are based on a biomass carbon content of 48% (w/w). BDL: below detection limit (0.01 mM).

|  | **30°C** | | **40°C** | | **Pooled Std Error** |
| --- | --- | --- | --- | --- | --- |
|  | **CBS4732** | **CBS11895** | **CBS4732** | **CBS11895** |  |
| D (h^-1^) | 0.10 ± 0.00 | 0.10 ± 0.00 | 0.10 ± 0.00 | 0.10 ± 0.00 | *± 0.00* |
| Reservoir glucose (g L^-1^) | 7.47 ± 0.02 | 7.49 ± 0.01 | 7.42 ± 0.02 | 7.43 ± 0.02 | *± 0.01* |
| Residual glucose (mM) | BDL | BDL | BDL | 0.02 ± 0.00 | *± 0.00* |
| Y_X/S_ (g biomass [g glucose]^-1^) | 0.49 ± 0.00 | 0.51 ± 0.00 | 0.46 ± 0.00 | 0.46 ± 0.01 | *± 0.01* |
| Y_X/O2_ (g biomass [g O_2_]^-1^) | 1.16 ± 0.02 | 1.35 ± 0.05 | 0.97 ± 0.00 | 0.99 ± 0.02 | *± 0.02* |
| RQ | 1.03 ± 0.00 | 1.05 ± 0.01 | 1.01 ± 0.00 | 1.04 ± 0.00 | *± 0.00* |
| q_Glucose_ (mmol [g biomass]^-1^ h^-1^) | -1.16 ± 0.02 | -1.08 ± 0.03 | -1.20 ± 0.01 | -1.21 ± 0.01 | *± 0.02* |
| q_CO2_ (mmol [g biomass]^-1^ h^-1^) | 2.78 ± 0.06 | 2.44 ± 0.07 | 3.27 ± 0.02 | 3.26 ± 0.02 | *± 0.04* |
| q_O2_ (mmol [g biomass]^-1^ h^-1^) | -2.69 ± 0.05 | -2.32 ± 0.08 | -3.23 ± 0.02 | -3.15 ± 0.02 | *± 0.04* |
| C_X_ (g biomass L^-1^) | 3.63 ± 0.02 | 3.84 ± 0.08 | 3.39 ± 0.04 | 3.41 ± 0.04 | *± 0.05* |
| Carbon recovery (%) | 98.2 ± 0.3 | 99.3 ± 1.2 | 100.5 ± 1.0 | 99.8 ± 1.0 | *± 0.8* |

Reported values are Mean ± MAD. Standard errors are reported in *italics* in the right column.

## Table S2: Temperature dependent maximum specific growth rates (µ_max_) of two wild-type *Ogataea (para)polymorpha* strains in aerobic shake flask batch cultures. Cultivation was done in synthetic medium with an initial glucose concentration of 20 g L^-1^. Means and mean absolute deviations (MAD) were calculated from two individual cultures and the standard error was calculated from the pooled estimate of variance across all culture samples from each strain. Logarithmic transformations of the data were applied to regularize the variance prior to pooling. Values for CBS4732 at 49°C and CBS11895 at 48 and 49°C were derived from cultures directly inoculated from glycerol stocks.

|  | ***O. polymorpha* CBS4732** | | | | | |
| --- | --- | --- | --- | --- | --- | --- |
|  | **30°C** | **37°C** | **40°C** | **45°C** | **48°C** | **49°C** |
| µ_max_, OD_660_ [h^-1^] | 0.34 ± 0.00  *±0.02* | 0.60 ± 0.01  *±0.03* | 0.66 ± 0.00  *±0.05* | 0.61 ± 0.01  *±0.03* | 0.37 ± 0.02  *±0.02* | 0.20 ± 0.03  *±0.01* |
|  | ***O. parapolymorpha* CBS11895** | | | | | |
|  | **30°C** | **37°C** | **40°C** | **45°C** | **48°C** | **49°C** |
| µ_max_, OD_660_ [h^-1^] | 0.40 ± 0.00  *±0.03* | 0.51 ± 0.00  *±0.03* | 0.59 ± 0.00  *±0.04* | 0.52 ± 0.01  *±0.03* | 0.28 ± 0.00  *±0.02* | 0.18 ± 0.00  *±0.01* |

Reported values are Mean ± MAD. Standard errors are reported in *italics* on the second line.
